# Risks of needlestick injuries in nursing homes for dependent seniors: myth or reality?

**DOI:** 10.1186/2047-2994-4-S1-P96

**Published:** 2015-06-16

**Authors:** P Fascia, N Khouider, A Fine, I Fourcade, S Laroche, A Savey

**Affiliations:** 1 Arlin Rhône-Alpes, Chu Lyon, Hopital H. Gabrielle, Lyon, France; 2 Diabetes Care, BD Medical, Le Pont de Claix, France; 3 CCLIN Sud Est, Chu Lyon, Hopital H. Gabrielle, Lyon, France

## Introduction

As part of the monitoring of needlestick Injuries (NSI), there is no data available on the risk of healthcare workers NSI in nursing homes for dependent seniors.

## Objectives

The objectives of this study are to identify risk situations of NSI, to estimate the occurrence risk of NSI and to evaluate the NSI management in nursing homes.

## Methods

This study took place from September 22^th^ to November 21^th^ 2014, by all nurses employees in nursing homes in the Rhône-Alpes region (France). This study is based on volunteering with 2 questionnaires available online: one for each institution and one for each nurse. The questionnaire for nurses is self-administered and anonymous. The descriptive analysis of regional data was performed using the software Epi-Info version 6.04.

## Results

Among 679 nursing homes in the Rhône-Alpes region (France), 163 participated to the study (24%). These nursing homes employed 1123 nurses, among whom 801 answered the evaluation (71%). 183 nurses reported a NSI event during their activity in nursing homes (22.8%), including 63 during the last 12 months of activity (34.4%), thus a NSI rate of 7.9% in the past year. The NSI occurred during: a subcutaneous injection (86 cases, 47.5%) including 60% when using a pen injector; a blood collection (59 cases, 32.6%) including 55.9% of capillary sampling; a subcutaneous infusion (18 cases, 10%). A third of these NSI occurred when removing the medical device. Thirty percent of nurses make the wound bleed. Nearly a third of nurses (29.3%) did not benefit from an expert opinion following the NSI and only a third (34.8%) received this expertise within 4 to 6 hours following the NSI.

## Conclusion

This study shows that nursing homes are also at risk of NSI, since nearly 1 nurse in 4 reports a NSI event. The use of pen-injector is one of the major causes of AES. Furthermore, the management of NSI appears as suboptimal in these institutions. This study should enable to raise awareness among all healthcare workers, and lead to the implementation of preventive actions for NSI in nursing homes.

## Disclosure of interest

None declared.

